# Context and mechanisms that enable implementation of specialist palliative care Needs Rounds in care homes: results from a qualitative interview study

**DOI:** 10.1186/s12904-021-00812-4

**Published:** 2021-07-22

**Authors:** Jane Koerner, Nikki Johnston, Juliane Samara, Wai-Man Liu, Michael Chapman, Liz Forbat

**Affiliations:** 1 Health Research Institute, University of Canberra, Canberra, Australia; 2 Faculty of Health, University of Canberra, Canberra, Australia; 3 Calvary Public Hospital Bruce, Canberra, Australia; 4 College of Business and Economics, Australian National University, Canberra, Australia; 5 Department of Palliative Care, Canberra Health Services, Canberra, Australia; 6 College of Health and Medicine, Australian National University, Canberra, Australia; 7 Faculty of Social Sciences, University of Stirling, Stirling, Scotland, UK

**Keywords:** Implementation, Care homes, Palliative care, Mechanism, Facilitation

## Abstract

**Background:**

Improving quality of palliative and end of life care in older people’s care homes is essential. Increasing numbers of people die in these settings, yet access to high quality palliative care is not routinely provided. While evidence for models of care are growing, there remains little insight regarding how to translate evidence-based models into practice.

Palliative Care Needs Rounds (hereafter Needs Rounds) have a robust evidence base, for providing palliative care in care homes, reducing resident hospitalisations, improving residents’ quality of death, and increasing staff confidence in caring for dying residents. This study aimed to identify and describe the context and mechanisms of change that facilitate implementation of Needs Rounds in care homes, and enable other services to reap the benefits of the Needs Rounds approach to care provision.

**Methods:**

Qualitative interviews, embedded within a large randomised control trial, were conducted with a purposive sample of 21 staff from 11 care homes using Needs Rounds. The sample included managers, nurses, and care assistants. Staff participated in individual or dyadic semi-structured interviews. Implementation science frameworks and thematic analysis were used to interpret and analyse the data.

**Results:**

Contextual factors affecting implementation included facility preparedness for change, leadership, staff knowledge and skills, and organisational policies. Mechanisms of change that facilitated implementation included staff as facilitators, identifying and triaging residents, strategizing knowledge exchange, and changing clinical approaches to care. Care home staff also identified planning and documentation, and shifts in communication. The outcomes reported by staff suggest reductions in hospitalisations and problematic symptoms for residents, improved staff skills and confidence in caring for residents in their last months, weeks and days of life.

**Conclusions:**

The significance of this paper is in offering care homes detailed insights into service contexts and mechanisms of change that will enable them to reap the benefits of Needs Rounds in their own services. The paper thus will support the implementation of an approach to care that has a robust evidence base, for a population under-served by specialist palliative care.

**Trial registration:**

ACTRN12617000080325.

**Supplementary Information:**

The online version contains supplementary material available at 10.1186/s12904-021-00812-4.

## Background

The number of older people living and dying in residential care homes (hereafter referred to as care homes) is increasing [1–3]; care homes are projected to be the most common place of death by 2040 [4]. Despite high levels of morbidity and mortality of people in care homes for older people, specialist palliative care is not uniformly provided.

Barriers to quality death and dying in care homes have many commonalities across nations, including limited staff knowledge [5], low staff confidence [6], insufficient staff training in palliative care [7], residents with complex needs [8], high turnover of staff [9], and resourcing and time constraints [10]. Competing demands on care staff result in lack of time to explore residents’ expectations for end of life care and advance care planning [11]. Where guidelines and education exist to support staff in providing palliative care in care homes, there are barriers in reaching the majority of staff [12].

Providing end of life support to care homes is an increasingly busy area of service development. Models such as ECHO [13], Gold Standard Framework [14], Six Steps to Success [15], the EU funded PACE work [16], and person-centred dementia care with the Namaste programme [17] offer staff training. However, those approaches rarely provide direct clinical care for people diagnosed as dying. Likewise, other models include elements of hospice outreach [18], anticipatory and advance care planning [19] and increasing organisational capacity and staff knowledge to deliver palliative care [20–22].

One model emerging in recent years called Palliative Care Needs Rounds (hereafter Needs Rounds) [23–26] combines many of these elements and has produced positive results. Needs Rounds lead to decreased hospital use [23, 24], increases in dying in preferred place [23], improvements in quality of death and dying [26], and higher staff self-reported confidence in adopting a palliative approach with residents [25].

However, even clinical models with a robust evidence base are not routinely taken up in practice. Consequently, the opportunities to substantially improve the quality of care for people living and dying in care homes is diminished. Implementation science developed as a field of enquiry to help maximise the potential for models to be adopted into practice. Implementation science suggests that by scrutinising the implementation contexts (at micro, meso and macro levels), mechanism of change, and outcomes of interest to organisations, it is possible to reduce the gap between research evidence and clinical practice [27]. These concepts of contexts, mechanisms and outcomes are derived from the PARIHS and iPARIHS frameworks [27, 28]. Context refers to the socio-political, legal and practical situation in which the intervention is delivered, at macro, meso and micro levels. Mechanisms of change refers to the activity (including processes) which triggers change, including reasoning (knowledge) and resource (such as people or equipment) mechanisms [29, 30]. Outcomes are operationalised as the change achieved as a consequence of adopting the intervention.

The current study adopts an implementation science framework to understand the contexts, mechanisms of change and outcomes for care homes engaging in Needs Rounds, to identify features which will enable other care homes to implement the approach thereby improving the quality of palliative and end of life care outcomes for residents.

## Methods

The data reported in this paper are drawn from qualitative interviews embedded within a stepped wedge randomised control trial (RCT) of Needs Rounds with 1700 residents across 12 care homes in Australia [24, 26]. The RCT delivered the Needs Round intervention (described below) over a period of 17 months. The primary outcome of interest to the trial was care home residents’ length of stay in hospital, secondary outcomes measured staff capability of providing a palliative approach, and resident quality of death and dying. Qualitative interviews were integrated into the design of the study, and are reported here.

### Aim

This study aimed to identify and describe the context and mechanisms that facilitate the implementation of Needs Rounds in care homes, and enable other services to reap the benefits of the Needs Rounds approach to care provision.

### Intervention

Needs Rounds are monthly hour-long meetings which discuss the bio-psycho-social needs of up to ten residents in care homes who have high symptom burden and who are at risk of dying without a plan in place. A checklist is used to guide discussions and outcomes [31]. They are chaired by a specialist palliative care clinician and attended by care home staff. Needs Rounds often trigger activities such as advance and anticipatory care planning, referrals to external organisations, prescribing and de-prescribing medicines, case conferences and bespoke clinical interventions. Needs Rounds include case-based education (that is, education based around one of the clinical cases or residents discussed), building staff knowledge and understanding of symptoms, recognising deterioration and discussing pharmacological and non-pharmacological treatments.

### Sample

All care homes in the Australian Capital Territory were invited to participate in the study, with the exception of those involved in the pilot (*n* = 4) and a site that was used for training (*n* = 1), leaving 21 care homes that were sent an initiation letter with information about the study. Care homes who participated in the RCT (*n* = 12) ranged in size from 42 to 165 residents. Some facilities were part of national chains, while others were privately owned. A purposive sample of staff from care homes using Needs Rounds were invited to participate, and 21 staff from 11 care homes were interviewed. The sample sought both senior and junior staff with a range of roles in the care home (for example managers, registered nurses [RNs], nursing assistants), who had attended Needs Rounds, were able to give informed consent, and were aged 18 or over.

### Data collection

Semi-structured interviews were conducted via one-off audio-recorded qualitative interviews. The questions were generated by the research team and built on questions used in the pilot work [25]. Interviews were conducted by a female academic with a decade of qualitative health care research experience, via face-to-face or phone interviews. About a third of participants had previous contact with the interviewer through collection of monthly quantitative outcome data from care homes in the RCT. The prior relationship was unlikely to have impacted the qualitative accounts generated, though it remains a possibility that accounts were bias in favour of the intervention due to social norms and face-saving. No differences however were observed between data generated from interviewees known to the research team, and those with no prior relationship. Participants were not provided with interview questions prior to the interview nor the resulting transcripts.

All sites had been using Needs Rounds for between six and eight months before staff were interviewed. Two facilities chose dyadic interviews, whereby two staff from the same care home were interviewed together. All other interviews were individual.

Interviews focused on views and experiences of care home staff regarding palliative care preparedness of care home before Needs Rounds, and impact of the intervention on staff and working practices (See Supplementary File for interview guide).

### Analysis

Interviews were audio recorded and transcribed verbatim by an independent transcriber and checked for accuracy. Two female health services researchers, who have PhDs in qualitative research, coded transcripts. Data were analysed by adopting a process of familiarisation,: coding, developing themes, indexing the data, synthesising across respondents and data interpretation to finalise key themes [32]. Analysis drew from abductive and inductive processes. Abduction refers to the analytic process of inferring from incomplete or limited data (notably not all sites participated in interviews and interview questions had not set out to investigate contexts, mechanisms of change or outcomes), which compliments the more traditional qualitative approach of induction, moving from specific data to creation of themes and interpretation. Analysis lent on implementation science theories, including the PARIHS and iPARIHS framework to examine the configurations of context, mechanisms of change and outcomes in order to determine a theory of what works for whom under what circumstances [27, 28]. The framework was used to conceptualise and organise the broad themes that arose in the interview data. Nvivo version 11 was used to store and organise data.

## Results

Twenty-seven staff were invited to participate; six declined or did not respond. Interviews were conducted with 21 staff members from 11 care homes (see Table 1 for demographics; the term ‘staff’ refers to this diverse group of people and roles). Seventeen were conducted face-to-face and four by phone. Nine hours and 39 min of interview data were transcribed; interviews lasted between 16:01 min and 53:36 min, with a mean of 52:18 min. Interviewee’s roles are not noted by the quotations in order to preserve anonymity of respondents.

**Table 1:** Demographic details of care home staff interviewees (*n* = 21)

**Role**	n
Care manager^a^	7
Facility manager	5
Deputy or assistant manager	2
Enrolled nurse	2
Team leader	2
Care assistant	1
RN	1
Clinical nurse specialist	1
**Age**	
21–30	7
31–40	4
41–49	4
51–60	2
61 +	4
**Sex**	
Female	17
Male	4
**Years of Experience**	
1–2	2
3–5	3
6–10	6
11–20	5
21 +	5

^a^management staff typically also held clinical roles

**Fig. 1:**
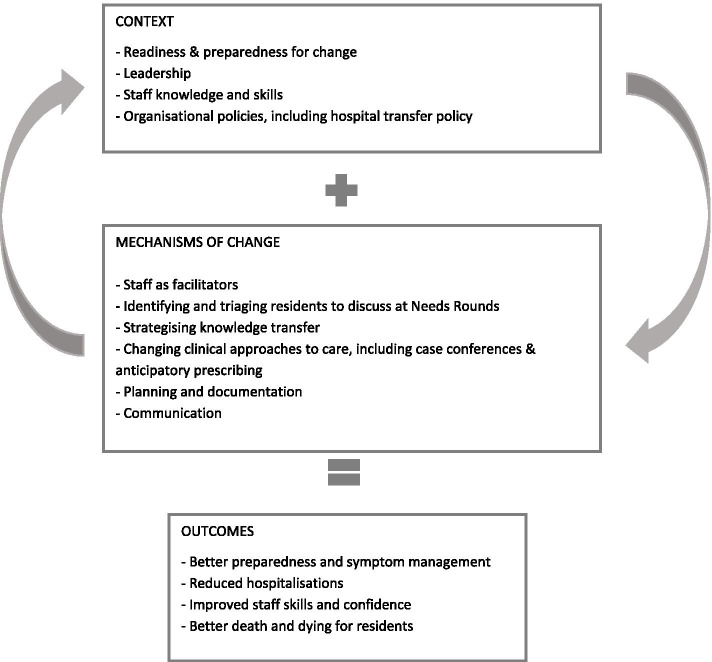
Needs Rounds Context, Mechanisms of Change & Outcomes

Drawing from the iPARIHS framework, we report major themes related to context, mechanisms of change and outcomes (see Fig. 1). Care home context contained subthemes of readiness and preparedness for change, leadership, staff knowledge and skills, and organisational policies. Mechanisms of change that facilitated implementation include sub-themes of staff as intervention facilitators, identifying and triaging residents to discuss at Needs Rounds, stategising knowledge transfer, and changing clinical approaches to care. Practical and concrete mechanisms of change that arose in response to Needs Rounds include contributions to planning and documentation, and shifts in communication. Outcomes are presented with sub themes of improvements in preparedness, staff skills and confidence, reduced hospitalisations, and better quality of death and dying.

### Context

Care home staff identified three influential contextual factors affecting implementation: (i) readiness and preparedness to change, (ii) leadership, (iii) staff knowledge and skills, and (iv) organisational policies, including for hospital transfer.

#### Readiness and preparedness for change

Care home staff reported varying levels of readiness and preparedness to change. Senior management engagement in wanting to improve palliative care was accompanied by strong support for implementing Needs Rounds. Some care homes were already thinking about how to create proactive discussions about end of life care, such as encouraging residents to put in place legally appointed alternate decision-makers and advance care plans when they enter the facility. While the majority of facility managers believed palliative care was important, most did not have concrete plans for implementation and felt that the staff team did not have clarity on how to provide palliative care.

#### Leadership

Leadership was an important contextual factor that ensured Needs Rounds were adopted. Care homes where managers and key personnel were strongly engaged with Needs Rounds signalled to staff the importance of taking time to undertake Needs Rounds and resulting actions.

*It’s good that all of our key people were really on board with it from the beginning… I think we embraced it from the beginning and it made – made it really easy then to come together as a group and discuss residents and the fact that we could see that they were actively dying and they were going to die in that next six month period. (Site 3, staff 1)*

##### Staff knowledge and skills

Staff reported that there was a great deal of variation in staff knowledge and skills regarding palliative care. Prior to using Needs Rounds, some staff equated palliative care to terminal care, while others had a broader view of palliative care. With the introduction of Needs Rounds improved staff knowledge and skill allowed for more residents to be cared for within the home rather than requiring transfer to hospital to manage symptoms:

*We didn’t have all the information and knowledge that we had prior. Now we’ve got all this information and knowledge we can use it within the facility rather than go, “Oh well we’ll just send you off to hospital.” (Site 3, staff 2)*

*We don’t need to send someone to the hospital, we don’t need to send someone to [the hospice] we can do it here we need to have confidence in the decisions we’re making as a team and in our skills. (Site 9, staff 2)*

#### Organisational Policies

Organisational policies often set the context for what care was permissible within the care home. Facility managers reflected that policies were often absent or inadequate in guiding how to identify and manage residents in need of palliative care. For example, palliative care policies included broad statements about the importance of palliative care, but little detail was provided on how to operationalise this.

Most care homes had formal or informal policies that mandated transfer to hospital if a resident fell, or showed a functional or physical decline. Needs Round meetings triggered discussion of whether policies were fit-for-purpose, and also reframing hospitalisation decision-making as being an appropriate element of advance and anticipatory care planning discussions with residents and families.

*It was ‘if in doubt, send out’ [for an ambulance]. That was actually the rule, whereas now that's completely changed and, you know, the theory is we go through the care plan, ‘what do they want’. ‘How did they want to die, where did they want to die?’ and that's definitely changed since we've been doing Needs Rounds. (Site 1, staff 1)*

Shifting the formal and informal policies around hospital transfers were therefore key contextual factors in achieving successful reduction of hospitalisations for residents observed in Needs Rounds studies.

### Mechanisms of change

Core mechanisms and processes that had changed to allow successful implementation of Needs Rounds were (i) staff as intervention facilitators (ii) identifying and triaging residents to discuss at Needs Rounds, (iii) strategising knowledge transfer (iv) changing clinical approaches to care, and (v) contributing to planning and documentation. Implementation also required shifts in (vi) communication to become a mechanism of change.

#### Staff as implementation facilitators

Implementation science puts considerable emphasis on the process of facilitation, and the role of facilitator. Our data indicate that both features were evident, with staff from both specialist palliative care and the care home engaged in facilitation activities:

*All of our key people were really on board with it from the beginning, and I think that was from having a good, healthy, strong relationship with [Nurse Practitioner from specialist palliative care] because she had been coming into the facility before. (Site 3, staff 1)*

#### Identifying and triaging residents

Establishing processes to identify dying residents to discuss was a mechanism that facilitated the avoidance of rushed decision-making of who to discuss minutes before Needs Rounds were due to start. Identification meant that the most appropriate residents were discussed, and minimal time was lost in deciding during the meeting.

Processes suggested by staff included: delegating the task of identifying residents to specific team members, emailing all staff prior to Needs Rounds, or leaving a ‘sign-up’ sheet in the staff room.

*We have the meetings on a Thursday we start thinking about “OK who’s been unwell? Who’s been to hospital? Who’s declining?” So we’re more aware of the signs rather than before well – I mean you’re aware but we’re looking for specific things now, which has helped us. (Site3, staff 2)*

#### Knowledge transfer

Interviewees reported that staff attending Needs Rounds had shared their new learning with other team members through written and verbal methods, as well as with residents and families. This had led to improved clinical knowledge and skills to care for the dying resident. Selection of staff to attend Needs Rounds also facilitates knowledge transfer. The knowledge gained in Needs Rounds case-based education was applied not just to that resident, but also to all residents affected by a specific symptom or issue.

*Really, we need to share it with everyone, because it’s not always the RN at the bedside that’s doing the care, we know that. It’s the care worker or the care supervisor. So they need to know as much as we [RNs] do…Yeah, then it becomes like a tool for education. (Site 3, staff 1)*

Methods used to facilitate knowledge transfer after Needs Rounds to all staff outlining the information discussed included emailing key points or meeting minutes, and writing into resident files.

*I take minutes for that meeting and then after we have the meeting I actually disseminate that to all the staff because we had an email address that goes to everyone. So I can, I can impart all of that information and everyone can read it. And then the care team management can actually copy and paste that information straight into their progress notes as well. (Site 3, staff 1)*

Verbal information sharing at hand over, at staff meetings, and at monthly training meetings were also used:

*Our knowledge-base has improved due to the Needs Rounds and the word of mouth. So when we have our AINs [Assistants in Nursing] and RNs that come to the Needs Round, they go and spread the word and give you a feedback to the other AINs and RNs and whatnot, and we do like a little teaching thing (Site 1, staff 1)*

Sharing information with families was also crucial, including on an individual level and through resident meetings with families. Engaging families in thinking about palliative care had improved recognising deterioration and dying:

*Every residents’ meeting I do talk about palliative care and dying and that if you haven't had a talk with me about deterioration and recognising what's going to happen, please contact me and if you do feel like your loved one is deteriorating, because sometimes it is normal for us to just not notice, because we do see them every day, that they do come and talk to us. (Site 1, staff 1)*

Ensuring staff across the care home were improving their knowledge, even if not directly attending Needs Rounds, was an important mechanism of change to improve communication with families and engaging them in anticipatory and advance care planning:

*We have a bigger knowledge and understanding of the whole palliative approach and also in the way we address the residents, especially the family members, because that’s one of the toughest, they want to understand and everyone of them, like from my experience, they will say “I don’t want that yet because he’s not dying.” Now that we have that Needs Round and we can easily explain to them and get them involved. (Site 10, staff 2)*

Identifying methods of disseminating the learning from Needs Rounds to the wider staff team, and filtering this through to conversations with families was an important element in successful implementation.

Although knowledge transfer operated as an important implementation mechanism of change, choice of which staff attended Needs Rounds was also important for their success.

Care homes required ways to identify staff to attend Needs Rounds, and this could be based on designated roles, or having a broad range of staff attending including carers and other allied health professionals such as occupational therapists. Other facilities were less strategic and invited whoever was available on the day to attend. Choice was driven by the local context:

*Really early on, where when we had the meetings there was like ‘OK, every senior person, plus anybody else that can come, will come to these meetings.’ And, you know, we want from each area we’d like two or three people to present, at least ten people at every meeting. (Site 3, staff 1)*

Both opportunistic and strategic approaches to choosing staff to attend worked for sites.

#### Changing clinical approaches to care

Needs Rounds often trigger clinical actions with residents, including case conferences held with residents, family members and general practitioners (GPs) to discuss palliative care and end of life choices, goals of care, symptom management and reviewing medicines. These clinical activities were mechanisms for improving care.

Prior to Needs Rounds, most care home staff did not have experience in conducting case conferences, and left organising and running case conferences to GPs and the specialist palliative care team. To assist staff to lead case conferences, one facility developed a template of questions to ask:

*As [staff] knowledge improves they’re also able to start doing a little bit more. So less reliant on, you know, having someone [from specialist palliative care] come and run the case conference for you (Site 3, staff 1)*

Needs Rounds led to improved processes for managing symptoms and ensuring anticipatory medications were in place. Proactive planning to identify changes in residents’ condition and possible trajectories needed to be in place so that anticipatory medications for pain or symptom management were charted and ordered:

*It's preparing, so having that medication in the cupboard. It's really important, our pharmacy is 35 minutes away, so that doesn't help. So when I first started here [Needs Rounds] hadn't gone in yet, so that was a year and a bit ago, three months ago, and we had a resident that was dying of pulmonary oedema and we didn't have the medication on hand. And it took me until 8.30 at night to go to a pharmacy somewhere to get the medication. Was that patient's health jeopardised and the way of their dying jeopardised? Yeah, it was. So that lesson then taught not only myself to be proactive when we need something really urgently. (Site 1, staff 1)*

#### Planning and documentation

Needs Rounds often trigger a range of activities for planning within care homes, as well as with other providers such as GPs. Planning activities identified by staff included documentation of goals of care, end of life choices, and increased proactive planning through case conferences with residents and families. At case conferences, possible trajectories and anticipatory care were discussed, and resident and families choices were documented in advance care plans. This meant that staff learnt to look for these documents when an acute event occurred or active dying symptoms developed, rather than relying on facility policy regarding hospital transfer. Staff reported that initially, this change created some confusion, for example, in the case of casual or weekend staff transferring residents to hospital despite the resident’s preferences for them to die in the care home and for their symptoms to be managed there:

*Now we have plans, we have actions, we can access medications for them. Their quality of life is a lot better when you have a plan in place. And the quality of death, when they’re pain-free and you don’t see them suffering I think it’s fantastic it should have been this way a long time ago. (Site 3, staff 2)*

*I think that’s another thing changing since Needs Rounds because advance care directive often was not done on time and we started looking for it when we really needed it so since Needs Rounds we discuss with the family and things and we always tell them we needed it as soon as possible so it’s in the place where when we need what they want. (Site 10, staff 2)*

Thus, providing a structure for anticipatory care conversations was an important mechanism that led to improved clinical care and decision-making.

#### Communication

Communication was both a mechanism for change and also was an improved outcome. Needs Rounds were used by staff as a mechanism to improve communication within the team, including generating ideas together. This led to increased communication in the care team overall:

*There’s been a lot of improvements with how we communicate with each other has really helped, we sort of bounce-off ideas from these meetings and we just all know so much more information and knowledge about how to care for end of life so we all have different ideas that we can discuss with each other about it. (Site 1, staff 2)*

Increased communication generated by engagement in Needs Rounds contributed to greater alertness to clinical concerns and discussing with families any identifiable changes in residents:

*We’re talking more frequently now; we’re checking more things like [our concerns] so I think staff are also getting benefit from they also coming to know okay this person’s having this, so we need to more alert and we need to inform the RN if more changes happen and things like that, so and some of the staff are quite good talking to the family as well. (Site 10, staff 2)*

Improving staff communication also operated as an advocacy mechanism, enabling staff to lobby for residents’ needs:

*We’ve been given the tools to speak out more. So never would have questioned my Director of Nursing about anything before. But now I’ve been taught that it’s not my Director of Nursing that’s dying, I’m not dying, my resident is and they deserve to have the best at this time. (Site 3, staff 1)*

Facilitating greater inter-agency and intra-agency communication were therefore powerful drivers for implementing Needs Rounds, and optimising outcomes for staff morale and resident wellbeing.

### Outcomes

Staff reported that Needs Rounds lead to better preparation that in turn reduced hospitalisations, and improved staff capability to deliver end of life care. The outcomes were multi-factorial including completed advance care plans, confidence to care for residents out-of-hours and improved communication with families:

*There's already a plan in place for that proactive treatment for death, is there. The next part of living is death and we're able to manage that no matter what time of the day. So there's always an RN, there is always a plan and that family are very well informed and are prepared for that phone call. (Site 1, staff 1)*

Planning and improved preparedness reduced unexpected symptoms arising, and allowed residents to be cared for in place.

*A lot of it was don't worry about it until the shit hits the fan, it really was but we're trying to pre-empt that, so when things do happen, because they're always unexpected most of the time, that it's okay, we can still respect the decision that you know Mr Smith wanted to die here. (Site 1, staff 1)*

Staff skills and confidence to communicate with residents, families, GPs and staff members about death and dying was improved.

*Much more confidence. Much more open. Transparent about talking about the whole thing, end of life, palliative care, what can we do to make this person more comfortable. I would say it’s a whole team. The whole team has moved in that regard. (Site 2, staff 1)*

This included more confidence to refer to specialist palliative care services, when needed.

*Before the Needs Rounds we were not calling- we were not involving the palliative nurse that much. It was only like you know few residents we think we cannot manage the pain at all, we just involved them. But now we got more confidence and now I know when to refer them. And we know you know they are there always like [name of nurse] is the one coming here and we know she’s always there for us. (site 11, staff 1)*

Staff felt that Needs Rounds led to better death and dying for residents.

*I think people now are dying in a – this sounds very odd, in a much better way, like, really – being pre-emptive having those conversations, preparing families, having that medication on hand so when it needs to happen it can straight away. And having people alert to the fact that there’s a significant – like there’s a deterioration and we need to do something about it and now I think that, that has led to some better outcomes and people dying in a really comfortable way and the families being at terms with it, before it happens, which means that they cope a whole lot better after the death. (Site 3, staff 1)*

The outcomes were therefore demonstrable in everyday practice for care home staff, as well as evident in the quantitative data [24, 26].

## Discussion

The data in this paper were derived from a qualitative study embedded within a large randomised control trial of Needs Rounds with 1700 residents, over the course of 17 months. The interview data describe the context, mechanisms of change, and outcomes which point to features which aid implementation of the Needs Rounds approach. The paper therefore compliments the quantitative outcomes of demonstrable improvements in hospitalisations and quality of death [24, 26] by reporting the features that enable implementation to be successful. The paper also offers a helpful contribution to the care home literature by identifying core factors affecting uptake of interventions, such as staff readiness for change, creating mechanisms to facilitate knowledge transfer and the role and process of facilitation.

The paper offers a unique contribution to the literature in documenting and describing the context and ways in which this Needs Round intervention is successfully implemented. It offers the starting point for generating an overall programme theory of change, which future research can explore and expand [33]. Future studies would helpfully examine perspectives of residents and relatives on the implementation, as well health and social care staff from beyond the care homes.

Care homes and specialist palliative care teams can use this paper to inform their adoption of Needs Rounds, preparing them to focus on the contextual factors and mechanisms of change highlighted in our data, to enable implementation.

Staff reported wanting to improve palliative care for residents as a strong motivator to implement change, but this needed to be supported by mechanisms, including time, processes to triage and identify residents to discuss, knowledge transfer, and clinical actions that arise including case conferences and anticipatory prescribing. Preparedness to adopt palliative care at organisation and staff level has been found to be associated with implementation potential in other palliative care studies [34, 35]. Time management, including staff absences and illness, has been identified as the biggest mediator of staff ability to devote time to new quality improvements on top of their regular work in care homes [15]. Involving staff early in improvement processes, providing ongoing telephone and email support throughout the program, and providing flexible support to individually tailor program to fit care homes are suggested as ways to address time as a barrier to implementation [15]. Change is needed at macro, meso and micro levels to enable the effective implementation of palliative care in care homes [36].

Education is also an important factor affecting successful implementation of programs in care homes; and processes to facilitate transfer of knowledge among staff should be prioritised with the appropriate health and social care, and facility policy frameworks to support these activities [37]. A systematic review of care home staff training emphasised the need for cultural change and ownership of the implementation within facilities [38]. Our study extended this notion of training into providing case-based education led by specialist palliative care clinicians, which was both individually well-received and also shared with the wider staff team.

Effective communication between specialist palliative care staff, care home staff, residents, families, and GPs are important components to quality care [39]. Needs Rounds support the need for communication and information sharing, as a knowledge expansion process, as well as a group decision-making process builds team confidence.

A scoping review of palliative care implementation strategies notes the need for interventions to support broader skills such as team-work [40]. Ensuring interventions focus on resident centred outcomes indicators are also needed [15]. Staff confidence is critical to improving communication about end of life in care homes, as self-efficacy is associated with increased communication ability [41] and capacity to implement new interventions [15].

Our findings indicate that there is appetite for care homes to implement Needs Rounds, evidenced by the approach being funded to embed the approach and sustain it beyond the study [42]. Needs Rounds have been taken up in rural settings and other jurisdictions with adaptations to implementation to suit their local context [43]. The transferability of the approach shows it can be successfully introduced to care homes in other settings, and once implemented can be continued remotely using telehealth [44].

This study reaffirms the need to consider systemic and contextual factors (including macro, meso and micro contexts) when implementing interventions to improve palliative care in care homes. Needs Rounds lead to better palliative care within care homes, by formalising and structuring in-reach from specialist palliative care programs into care homes. Factors such as the organisational and regulatory context of care home facilities, acute hospital proximity, and availability of hospital and community liaison teams all contribute to the local environment which impact implementation potential and success. A systems-informed approach to implementation bolsters likelihood of success for implementation to take place [45].

Our qualitative data had limitations, with no interview questions investigating the impact and role of multi-agency working, facilitation, changes in commissioning, or wider health and social care policy changes. Needs Rounds were implemented within this study utilising research funding, under trial conditions and within one metropolitan setting. Contextual differences in other settings may reveal different implementation needs for ideal outcomes, particularly over longer time periods, and this will require additional study. Use of Needs Rounds beyond Australia requires further development including in both majority and minority world countries with private and public health systems. As described elsewhere, the conceptual overlaps and operation of contexts, mechanisms and outcomes can be complex, and operate on a continuum [29].

## Conclusion

This study identified staff and organisation readiness, time, organisational policies, documentation, transfer of knowledge and collaboration as factors that facilitate implementing of Needs Rounds. The contextual features and change mechanisms in the qualitative data crystallise common factors which have potential to be applied to other care home interventions to improve implementation.

Needs Rounds can be adapted to meet local contexts, to meet the palliative care needs of older people in care homes in contexts sharing similar features.

## Supplementary Information

**Additional file 1.**

## Data Availability

The datasets used and or analysed during the current study are available from the corresponding author on reasonable request.

## Abbreviations

AIN: Assistant in Nursing
GP: General Practitioner
RCT: Randomised Control Trial
RN: Registered nurse
