# Supplementary material for: Context and mechanisms that enable implementation of specialist palliative care Needs Rounds in care homes: results from a qualitative interview study
Source: BMC Palliat Care. 2021 Jul 22;20:118. doi: 10.1186/s12904-021-00812-4 (PMC8299598; doi:10.1186/s12904-021-00812-4)
Supplement: Supplementary file 1 — Additional file 1. [file 12904_2021_812_MOESM1_ESM.docx]

**Interview topic guide**

1. Tell us about your facility. Specifically:
   1. How many Registered Nurses (RNs)?
   2. What RN cover is there overnight and weekends?
   3. Anything else to note on staffing, e.g. in-house GP?
   4. Before we started needs rounds, what was your organisational policy on hospital transfers? (e.g. ‘always transfer if x occurs’).
   5. How ‘palliative care ready’ was your facility?
   6. Are you part of a bigger chain of facilities, and if so, how does that influence your palliative care provision here?
2. What has it been like using Needs Rounds as the new model of care?
   1. Has it made any difference, and if so, in what ways?
3. Since Needs Rounds was introduced, has there been any impact on how staff document and discuss end of life care of residents? If so, in what way/s?
4. Has the Specialist Palliative Care Clinician involvement changed your/ staff’s knowledge about caring for people at end of life? If so, what and how has this impacted care? Or impacted you?
5. What impact has Needs Rounds had on your/staff confidence in identifying people who are likely to die in the next few months now?
6. Do you/staff feel more confident about caring for people who are dying now? If so, why?
7. Has this new model impacted your/staff confidence in talking with relatives about death and dying?
8. What impact has the new model had on your/staff confidence in having conversations about planning for death including advance care planning and preferred place of death?
9. What impact has the new model had on your/staff confidence to run a multidisciplinary case conference?
10. Since the Specialist Palliative Care Clinician became involved, has there been any impact on managing symptoms for residents? (e.g. pain, constipation, mouth care)?
11. What have you/staff learnt about transferring residents to hospital versus keeping them at the care home?
12. What has the impact been of having a nurse prescriber involved in resident care, regarding access to medicines and symptom control.
13. What impact has the trial had on your/staff confidence to use medicines at end of life
14. Has the new model made any difference to staff morale?
15. Has the new model made any difference to residents’ quality of life?
16. Has the new model made any difference to residents’ quality of death?
17. What was easy/hard for your facility in adopting the new model?
